# Investigating the sociodemographic profile and health-related outcomes of chronically ill homeopathic patients: results from an observational multi-centered study in Bulgaria

**DOI:** 10.1186/s12889-019-7914-7

**Published:** 2019-12-09

**Authors:** Desislava Vankova, Iskra Kapincheva

**Affiliations:** Department of Social Medicine and Healthcare Organization, Medical University of Varna, Marin Drinov str. 55, 9002 Varna, Bulgaria

**Keywords:** Chronically ill patients, Homeopathy, Bulgaria, Public health

## Abstract

**Background:**

The Complementary and Alternative Medicine (CAM) field in Europe has been a focus of research developments and public health policy changes for years. However, these processes are still in their infancy phase in Bulgaria. In this paper, homeopathy is the investigated CAM-modality at a Bulgarian context. The aim is threefold: 1) to outline the sociodemographic profile of the chronically ill adult patients (≥18 years old) who choose homeopathic medical treatment (HMT); 2) To identify the patients’ sources and needs of information about homeopathy, and the reasons to use HMT; and 3) to measure health-related outcomes in patients who have visited homeopathic clinics to look for HMT of their chronic conditions.

**Methods:**

A cross-sectional observational multi-centered study (≥18 years old, *n* = 211) was conducted between June 2016 to Dec. 2017 in Bulgaria. Potentially eligible participants for the study were all chronically ill patients who had been receiving HMT for a year or more and had visited the homeopathic clinics for a follow up within the study period. The EQ-5D-3L instrument was applied with an additional questionnaire on sociodemographic and health related data.

**Results, discussion and conclusions:**

The study results outlined the country specific sociodemographic profile of the chronically ill homeopathic patients in Bulgaria: they are predominantly female, with higher education and at the age groups between 30 and 50.

The EQ-5D-3L version, was applied to measure patients’ health-related quality of life and to promote the use of a standardized generic instrument as a complementary and reliable scientific tool to assess the patient-reported outcomes of the homeopathic patients. Regarding the reasons of choice: the participants choose HMT because it is safe and mild, to avoid excessive antibiotic use, because the conventional treatment was not successful, etc. The study confirms the social demand for more scientific information about homeopathy. Participants stated that the medical universities’ curriculum should keep pace with the modern CAM-developments. A key message is that the future of the successful chronic disease management is in the integration of the conventional and CAM-modalities and these processes should be facilitated through public health regulations, education and research. The presented study is a supportive action in this direction.

## Introduction

The Complementary and Alternative Medicine (CAM) field in Europe has been a focus of dynamic research developments and public health policy changes for years [1–6]. However, these processes are still in their infancy phase in Bulgaria, a former communist country, which has since 2007 been a European Union (EU) Member State.

One of the most pressing findings of the EU CAMbrella Project [7, 8] was the lack of sufficient, reliable and comparable data about CAM in Europe. The 2019 World Health Organization (WHO) report on traditional and complementary medicine [9] addresses this gap in credible and official data. Bulgaria is among the WHO Member States that has acknowledged the use of CAM and has formally developed public health regulations for the non-conventional medical methods, which incorporate some CAM-modalities [10]. However, a more comprehensive country summary is missing [9]. Furthermore, Bulgaria was not included in the EU CAMbrella project and is still unchartered territory on the map of the European CAM territory [11]. Nationally, there is not sufficient data about the use of CAM modalities, which is an obstacle for the adequate integration of CAM into Bulgarian healthcare.

Research shows that the use of CAM in the former Soviet countries has been growing but a country-specific investigation is needed [12]. A recent study on 12-month prevalence of traditional and CAM provider use among adults from 32 countries, shows that Bulgaria, Poland and Slovenia are with the lowest CAM-provider use prevalence (10%, mean 26.4%) [13]. However, the field of CAM is a heterogeneous and culturally diverse concept that comprises many curing methods and systems [6]. For example, In Bulgaria the CAM-modalities, which are native for Asia, as acupuncture and Ayrveda, are popular with the term “Eastern medicine” [14]. The term “folk medicine” (“narodna meditsina”, in Bulgarian) is the most widely used to define the CAM modalities that are native for Bulgaria. Folk medicine in the country is the “oral collection of all curing traditions (methods and materials – herbs etc.) which are sustainably transmitted from generation to generation” [15]. The CAM-modalities with European origin as homeopathy and naturopathy are gaining popularity but these processes are still under-investigated. Consequently, the reported results will be both socially and politically relevant for the Bulgarian society but also beneficial for the international audience because they fill in a gap in research information regarding CAM.

In this paper, homeopathy or the “similia similibus curentur” medical approach [16] is the investigated CAM-modality at a Bulgarian context. The country has long traditions in homeopathy as a therapeutic method. It was formally introduced in the country during the late nineteenth century. These traditions have been interrupted in 1944 when homeopathy was forbidden and revived in 1989 alongside with the social and political changes. Since then homeopathy has quickly gained popularity in Bulgaria. Generally, public health regulation which frames the practice of homeopathy and the use of homeopathic remedies meet the international standards. The existence and practice of homeopathy is legally supported by: 1) The Law on Medicinal Products and Pharmacies in Human Medicine [17], according to which homeopathic preparations are complete medicaments and are subject to official registration in Bulgaria; 2) The Health Law, in the Chapter “Unconventional Methods for Improvement of Individual Health”, Art. Article 167, which states that homeopathy may be practiced only by masters in medicine and dentistry [18] and defines the non-conventional methods. Among these methods is homeopathy. Herewith is adopted the term HMT that encompasses the following characteristics: individualized homeopathic care provided by a medical doctor with a standard qualification in homeopathy. In HMT the primary homeopathic consultation involves intensive listening and questioning of the patient alongside the medical examination. Using the frame of the homeopathic interview the specialist inquires and detects the whole picture of the state of patient’s body and mind, which allows the homeopath to prescribe the appropriate remedy. The follow-up visits are scheduled individually and for the chronically ill adult patients this entails a long period of time. Homeopathy is not integrated in the national health insurance system and the payments are only out-of-pocket. Nevertheless, the number of patients who seek HMT is growing but there is no relevant national data on the prevalence of homeopathy use in Bulgaria. The presented study has made the first steps to fill this data gap and to create ground for future homeopathic research integrated in the European CAM paradigm. Nationally, homeopathy is no more only a clinical practice but a public health issue at stake. Globally, homeopathic medicine attracts the public health interest for many reasons, such as: 1) scientific analyses which investigate the beneficial role of IHT in the long-term care of patients with chronic diseases and the cost-effectiveness of IHT use in general practice [19, 20]; 2) the use of homeopathy is steadily rising. For example by approximately 15% among the US adults – from 1.7% in 2002 to 2.1% annual prevalence rates of homeopathy use [21], and in South America, Asia and in Europe homeopathy is used by an increasing number of their citizens [8, 22, 23]; 3) the cost-effectiveness of homeopathy is viewed by analysts as an opportunity to lower pharmaceutical spending [24].

Worldwide, the reasons to choose homeopathy as a therapy are miscellaneous. In Europe, motivation to use CAM therapies can be divided into three main groups: to treat chronic diseases, to deal with acute illnesses and to maintain good quality of life [25]. Therefore, the issue of improving health outcomes in individuals with chronic medical conditions is an important public health topic. There is growing scientific evidence that homeopathy is improving quality of life in long-term care [26, 27].

Quality of life is a term used to describe how a person feels and functions in his/her everyday life. Particularly, health-related quality of life (HrQoL) is concerned with how well people are able to function and how they feel about physical, mental, and social dimensions of their lives [28]. HrQoL is one of the health outcomes recommended by the WHO for health monitoring of chronically ill patients e.g. patients who have diseases which require a long duration of care and show generally slow progression as a result of a combination of genetic, physiological, environmental and behavioral factors [29].

In Bulgaria, homeopathy is used mainly in out-patient clinics, which are the study settings of the presented research. The aim of this study is to present country-specific new evidence (sociodemographic and patient-reported outcomes) and thus to contribute to the international scientific development of the CAM field.

The **aim** is threefold: 1) to outline the sociodemographic profile of the chronically ill adult patients (≥18 years old) who choose HMT; 2) To identify the patients’ sources and needs of information about homeopathy, and the reasons to use HMT; and 3) to measure the HrQoL in patients who have visited homeopathic clinics to look for an HMT of their chronic conditions. The idea is to take a snapshot of the current situation in the country, related to the use of homeopathy for chronically ill patients; to assess the state of their HrQoL and to identify relevant topics for future research.

## Methods

### Study design and sampling

**Fig. 1:**
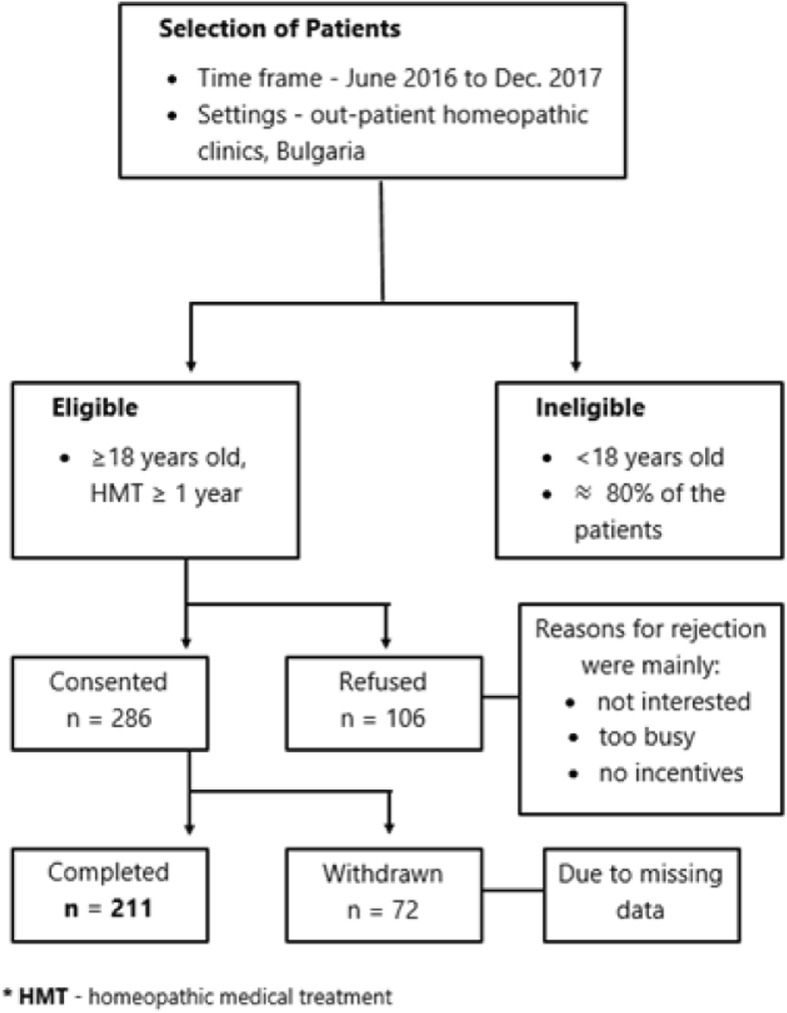
Flow diagram of sampling process and patient enrollment

A cross-sectional observational multi-centered study among chronically ill homeopathic patients was conducted between the second half of 2016 to Dec. 2017 in Bulgaria. The study settings were four large out-patient homeopathic clinics in the towns of Burgas, Varna, Veliko Tarnovo and Sofia, which treat patients from all over the country. The head physicians working in these homeopathic clinics were certified in homeopathy by the European Committee for Homeopathy (ECH), have more than twenty years of experience as homeopaths, practiced more than 30 h per week and are lecturers in homeopathic medicine. Potentially eligible participants for the study were all chronically ill patients who had been receiving HMT for a year or more (≥18 years old) and visited the homeopathic clinics for a follow up within the study period. With the above inclusion criteria the sample was selected as follows (Fig. 1): over the study period (the second half of 2016 to Dec. 2017), every chronic adult patient was consecutively invited to participate upon their regular visit to their homeopathic doctor for a follow up. A member of the outpatient setting nurse or secretary, approached every eligible chronically ill patient to propose participation in the study. The response rate varied between 65 and 80% depending on the clinical setting. The reasons for rejection were mainly: not interested and too busy. At the end of the study period 286 patients agreed and filled in the provided questionnaire, of them 75 questionnaires were excluded from the analysis because of too much missing sociodemographic and HrQoL information. Finally, the sample size reached 211 patients whose questionnaires have been accepted as informative and eligible for analysis. It is important to underline that around 80% of all the patients in the participating homeopathic clinics are children (< 18 years) who are not included in the study. As the study was observational, were included patients with conventional diagnosis from the whole clinical spectrum, there were no preliminary restrictions on the length of the consultation and the prescribed homeopathic medicines. All study participants provided written informed consent.

The study protocol has received approval by the Commission of Ethics in Scientific Research, Medical University - Varna, Bulgaria (Protocol № 57, Year 2016; Final report accepted with Protocol № 75/07.06.2018).

### The instrument

Homeopathy aims to cure the patient not only the disease. Therefore, HMT relies on improvement in overall well-being. These can be measured by a quality of life instrument, which could be a complementary tool during a homeopathic treatment. The European Quality of Life Five Dimensions (EQ-5D) instrument is such a reliable measure of patient-reported outcomes [30]. In this study the 3 Level version- EQ-5D-3L, paper version was used and the Bulgarian version was provided by the EuroQol Research Foundation, especially for the project. The EQ-5D-3L is a generic self-administered questionnaire which defines health in five dimensions including mobility, self-care, usual activities, pain or discomfort, and anxiety or depression. Each dimension has three levels ranging from “no problem” to “extreme problem”. Furthermore, EQ-5D-3L consists of two components. The first part is a questionnaire, a descriptive element with the five dimensions. Respondents rating their health status are asked to select the level of dimension, which describes at best their “health today”. On the second part, respondents record their self-rating of health on a 20-cm Visual Analogue Scale (VAS -“Thermometer” type) anchored by “100- denoting as best imaginable health state” and “0 - denoting as worst imaginable health state.” The validity and reliability of the EQ-5D-3L questionnaire have been tested extensively [31–33].

The study protocol was designed to evaluate HrQoL of chronically ill patients who seek HMT due to them having different diagnoses which was the reason a generic (EQ-5D-3L) was applied and not a disease-specific measurement instrument [34]. The protocol involved only the chronic patients who had been receiving HMT for a year or more (≥18 years old, number: 211). The main outcome measure was the current self-reported HrQoL. From this group, the data about the sociodemographic profile of the chronically ill adult patients (> 18 years old) who chose HMT, was obtained. The patients were asked to write information about use of other CAM or conventional therapies, the duration of the homeopathic treatment, the sources and needs of information about homeopathy and the reasons to choose HMT. Finally, change of the severity of complaints after the homeopathic treatment (the patient evaluates his/her health change after the homeopathic treatment in 4-points rating scale from “my health is ‘much better’ to ‘worse’”). There was a place at the end of the questionnaires for comments by the patients about the reasons for choosing HMT and if they would like to add something, related to their previous answers in a narrative manner. The design of the study and the questionnaire were presented at the First European Congress for Homeopathy (Vienna, 17–19.11.2016) [35]. After a written informed consent, the patient was included in the study. Patients had been included until a defined final date, namely 25 of December 2017. The questionnaire was completed by patients independently of their physician.

Initially, the HrQoL-instrument was piloted with 35 homeopathic patients to test the face validity. The questionnaire was pre-tested to decide on the number of additional questions (aiming at appropriate length of about 10 to 15 min) and to improve the wording.

### Data analysis

Descriptive statistics were used to identify the basic sociodemographic features of the patients included in the study and to analyse the HrQoL data frequencies; mean and standard deviation (SD) were used for the VAS-data analysis. Statistical analyses include also application of correlation analysis to measure the direction and strength of the linear association between the length of HMT and HrQoL. The analysis was carried out using the statistical software package IBM SPSS Statistics Version 23.

## Results

### The sociodemographic profile

The characteristics of the group, summarized in Table 1 (*n* = 211, > 18 years old), can be regarded as a sociodemographic profile of the chronically ill adult patients (≥18 years old) who chose HMT in Bulgaria. Analysis of the group showed that 81% (171) of the patients who seek HMT for their chronic conditions were women and 73.93% (156) of the patients have higher (university) education. Even though the professional profile is heterogeneous (IT-specialists, stewardesses, traders, legal professionals and many others), 18.96% (40) of the patients are working in the healthcare sector as physicians, nurses, veterinary, medical representatives in pharmaceutical companies, physiotherapists, speech therapists, social workers, dentists etc. Most patients - 80.09% (169), are between the ages of 18 to 44 years.

**Table 1:** Sociodemographic characteristics of the sample (*n* = 211, ≥18 years old)

Gender	Percentage (Number)
Female	81% (171)
Male	19% (40)
Age	
18–24 years	3.32% (7)
25–29 years	7.11% (15)
30–34 years	14.69% (31)
35–39 years	31.75% (67)
40–44 years	23.22% (49)
45–49 years	8.53% (18)
50–54 years	4.27% (9)
55–59 years	4.27% (9)
60–64 years	1.42% (3)
> − 65 years	1.42% (3)
Education	
Higher (University)	73.93% (156)
Secondary	22.27% (47)
Primary	3.8% (8)
Profession	
Health-related	18.96% (40)

### Overall EQ-5D-3L – status and health status change after a year or more with homeopathic treatment

The overall HrQoL results are presented in Table 2. As shown in the table, patients without any problem in all five dimensions represent 29.5% (*n* = 65) of the respondents, whereas only 6.2% (*n* = 13) reported any extreme problem in at least one dimension. Extreme problems were most often recorded in the “anxiety/depression” dimension. The mean state of health on VAS is 75.31 (SD ± 18.227).

**Table 2:** Share of respondents in each dimension (5D) and level (3 L), and frequency of reported problems, non-age standardized; VAS-mean of the study

Level of problem (3L)	Mobility		Self-care		Usual activities		Pain/ discomfort		Anxiety/ depression		VAS
	Number	%	Number	%	Number	%	Number	%	Number	%	
No problem	179	84.8	204	96.7	174	82.5	76	36.9	65	30.8	
Some problem	32	15.2	7	3.3	36	17.1	132	62.6	133	63.0	
Extreme problem	0	0	0	0	1	0.5	3	1.4	13	6.2	
Mean state of health - VAS											75
Other studies (share of respondents with problems, for comparison reasons)											
All EQ-5D-3L		16.6		4.5		14.6		37		24.6	78
EQ-5D-3L-Europe		17.1		4.9		14.6		37.9		24.7	77
EQ-5D-3L-Bulgaria		34.2		23.3		28.7		57.3		56.8	70

The key inclusion criterion for the study was “HMT longer than a year”. The highest proportion of chronically ill patients in the study 64% (*n* = 137) had been using HMT for longer than 3 years, 13.7% (*n* = 29) had been using HMT for longer than 1 year, while 22.3% (*n* = 47) use HMT for more than a year but less than 3 years. With an additional question the patients were given the opportunity to assess their overall health status change after a year or more with homeopathic treatment in a 4-steps rating scale from ‘much better’ to ‘worse’. It is evident from the patients’ answers that 33.6% (*n* = 71) assess their health status after a homeopathic treatment as ‘much better’, 42.7% (*n* = 90) as ‘better’, 20.4% (*n* = 43) as ‘no change’, and only 3.3% (n = 7) patients assess their health status as ‘worse’ than a year ago.

Nearly 3/4 of the patients (*n* = 156, 73.9%) were treated with other therapies, including conventional remedies before they sought HMT and started to take homeopathic medicines. At the moment of the study conduct, more than a quarter of the patients (*n* = 64, 30.3%) are using homeopathic remedies as complementary: to the conventional (*n* = 39, 18.5%) or to other forms of CAM-therapy, like Traditional Chinese medicine, Schussler therapy, folk medicine etc. (*n* = 25, 11.8%). Only 69.2% (*n* = 146) are treated with homeopathic medicines as monotherapy.

### Sources and needs of information related to homeopathy. Reason to choose HMT

Further sources and needs of information related to homeopathy have been investigated. More than 7 in 10 patients (*n* = 154, 73%) have learnt about homeopathy as a therapeutic method from friends and acquaintances; only 5.7% (*n* = 12) from their family doctor or other medical specialist, 17.1% (*n* = 36) from books and media (TV, radio, internet). Obviously, some patients (4.3%, *n* = 9) have learned about homeopathy from more than one channel of information. More than 6 in 10 patients (*n* = 132, 62.6%) need more information about homeopathy.

At the end of the survey, the patients had the opportunity to add comments, which could give additional information related to the reasons of homeopathy as a choice etc. Herewith, a brief summary of the finding is presented: the comments were ranging from “I would not go back to conventional medicine” to “The treatment of diseases is unthinkable without synergy of both medical e.g. conventional and complementary methods.” The patients shared that they are thankful to homeopathy and their homeopathic doctor: “I am now another man, lively and vibrant.”; “Since I have been using homeopathy I feel reborn. I would love to enrich my knowledge about homeopathic treatment”, “Homeopathic treatment brought me into remission. This is a miracle. After 10 years of corticosteroids, I no longer drink them and feel much better. I suffer from rheumatoid arthritis and Dercum’s disease.”,” I am happy that I have the opportunity to choose homeopathic treatment for myself and for my family.” And yet patients insisted on more relevant information: “There is a need for promoting the benefits and advantages of the homeopathic method.”, “People need to know that treatment is mild, effective and harmless”. Additionally, some participants stated that every physician needs to be educated regarding CAM and to keep pace with the modern CAM-developments.

Among the reasons that the patients point out for the use of HMT are: HMT is a way of stimulating the immune system; HMT is effective and safe; no result from the conventional treatment; we use it not only for our chronic condition but for the whole family. There are also some culturally specific characteristics. Many patients consider and use interchangeably the terms “conventional medicine” and “traditional medicine”.

## Discussion

The aim of the presented cross-sectional observational study was derived from and was inspired by the CAMbrella EU project and the growing scientific evidence related to homeopathy [36]. Worldwide, the number of people who seek CAM-therapies and particularly for HMT is growing and the research community needs to reflect these trends [8, 21–23].

The presented study provides a country-specific sociodemographic profile regarding HMT and chronically ill homeopathic patients. The study settings were large out-patient homeopathic clinics in four of the biggest cities in Bulgaria (Burgas, Varna, Veliko Tarnovo and Sofia), which treat patients from all over the country. Therefore, it can be suggested that the study is nationally representative and that the sociodemographic profile is relevant for the country. The reported results are consistent with the characteristics of other international studies [19, 21, 22, 37], which found that the adult homeopathic patients are predominantly female, with higher education and at the age groups between 30 and 50. Male patients who were invited to participate in this study often refused to sign the informed consent and therefore did not take part in filling in the study questionnaire. However, this information does not change the fact that more women are seeking HMT than men.

Generally, one of the ultimate goals of every HMT is to improve and maintain the quality of life of the patient. The standardized EQ-5D-3L version, created by the EuroQol Group, was applied. The wholistic and individual approach to health are characteristics both for HMT and for HrQoL-measurements. The idea was to promote that a standard generic instrument could be a complementary and reliable scientific tool to measure the patient-reported outcomes of chronically ill homeopathic patients. From an international perspective, the representativeness of this study and the applied standardised EQ-5D-3L instrument provide reliable and comparable data about homeopathy as a CAM-modality. As different as countries are, regarding their health care systems, future research on HrQoL of CAM-patients with a standardised tool, like the EQ-5D-3L questionnaire, could be a scientific basis for international comparisons and relevant source for public health policy formulation related to CAM. Hence, investigating HrQoL in CAM and particularly in homeopathy is a relevant scientific way to fill-in the data-deficit regarding “similia similibus curentur” medical approach [38, 39].

The levels of self-reported health problems of the patients in this study are even lower than the levels reported in a previous population cross-sectional study in Bulgaria [40]. Generally, the patients from the study cohort (*n* = 211, > 18 years old), reported high levels of HrQoL. Moreover, the longer the patients have received HMT the higher they self-rated their health status (VAS scores). There is a positive correlation between longevity of homeopathic treatment and VAS-self-rated valuation (R = 0.171; *p* = 0.013). The patients who use HMT more than 3 years report higher HrQoL in all five dimensions (mobility, self-care, usual activities, pain/discomfort, anxiety/depression) but the results are not statistically significant (R = 0.120; *p* > 0.08). However, at the moment of the study conduct, more than a quarter of the patients (*n* = 64, 30.3%) are using homeopathic remedies as complementary to the conventional or to other forms of CAM-therapy, like Traditional Chinese medicine, Schussler therapy, folk medicine etc.; while 69.2% are treated only with homeopathic medicines. Consequently, the higher levels of HrQoL could be a result of the HMT but further research is needed to prove correlation and causality.

Finally, the study confirms the social demand for more scientific information about homeopathy. Many participants shared the idea that not only do the patients need more information about homeopathy but the medical doctors too. The medical universities’ curriculum should keep pace with the modern CAM-developments, which means that studying non-conventional medical methods at universities should be mandatory, not recommended because medical specialists and healthcare professionals need to be competent regarding CAM-modalities. The fact that 18.96% (40) of the patients are working in the healthcare sector as physicians, nurses, veterinary, medical representatives in pharmaceutical companies, physiotherapists, speech therapists, social workers, dentists etc. indicates that the acceptance of HMT among the professional community is growing. We believe that the future of the management of chronic diseases is in the integration between the conventional and CAM modalities and these processes should be facilitated through education and research.

## Conclusion

With all the above analyses in mind, the presented research successfully reflected on implementation of approaches directed towards: 1) a broader understanding and social promotion of homeopathy as CAM in Bulgaria; 2) a greater appreciation of public health importance of evidence-based homeopathy; 3) a growing interest in using HrQoL-instruments as patient-reported outcomes in the monitoring of chronic disease therapies including CAM and particularly homeopathy. Subjective assessment of the health status is a way “to hear the voice” of the patient. Similar scientific investigation in Bulgarian and regional context has not been conducted and reported to our knowledge. This fact demonstrates the social implication and public health importance of the current study. Even though, there is an increasing demand and use of homeopathic care in Bulgaria, the related research is underdeveloped and needs reinforcement [41]. There should be further research because HMT-demands are expanding and need adequate local and regional public health policies support, which in turn needs relevant scientific evidence [42]. The presented research is a supportive action in this direction.

## Limitations

One of the limitations of the study comes from the fact that it is cross-sectional and gives us only the current situation and not the therapeutic process evaluation in dynamics. The major limitation is that the impact of homeopathic use is difficult to be separated from other therapies. Recall bias are also possible because the patients are supposed to recall facts about their health status 1 year or even 3 years ago. The major organizational limitation comes from the fact that homeopathy is not publicly funded and it is very difficult to organize a study with patients who pay out-of-pocket, because they are not always willing to participate. Therefore, additional human and financial resources are required for a successful project continuation.

## Data Availability

The datasets used and analysed during the current study are available from the corresponding author on reasonable request.

## Abbreviations

CAM: Complementary and Alternative Medicine
ECH: European Committee for Homeopathy
EQ-5D-3L instrument: European Quality of Life Five Dimensions Three Levels instrument
EU: European Union
HMT: Homeopathic medical treatment
HrQoL: Health-related quality of life
SD: Standard deviation
VAS: Visual Analogue Scale
WHO: World Health Organization
